# Comprehensive genetic characteristics of dystrophinopathies in China

**DOI:** 10.1186/s13023-018-0853-z

**Published:** 2018-07-04

**Authors:** Peipei Ma, Shu Zhang, Hao Zhang, Siying Fang, Yuru Dong, Yan Zhang, Weiwei Hao, Shiwen Wu, Yuying Zhao

**Affiliations:** 1 Department of Neurology, the General Hospital of Chinese People’s Armed Police Force, Beijing, China; 2 Department of Magnetic Resonance, the General Hospital of Chinese People’s Armed Police Force, Beijing, China; 3 Department of Precision Medicine Laboratory, the General Hospital of Chinese People’s Armed Police Force, Beijing, China; 4 Research Institute of Neuromuscular and Neurodegenerative Diseases and Department of Neurology, Qilu Hospital, Shandong University, Jinan, Shandong China

**Keywords:** Dystrophinopathies, Duchenne muscular dystrophy, Becker muscular dystrophy, Mutation spectrum

## Abstract

**Background:**

Dystrophinopathies are a set of severe and incurable X-linked neuromuscular disorders caused by mutations in the dystrophin gene (*DMD*). These mutations form a complex spectrum. A national registration network is essential not only to provide more information about the prevalence and natural history of the disease, but also to collect genetic data for analyzing the mutational spectrum. This information is extremely beneficial for basic scientific research, genetic diagnosis, trial planning, clinical care, and gene therapy.

**Methods:**

We collected data from 1400 patients (1042 patients with confirmed unrelated Duchenne muscular dystrophy [DMD] or Becker muscular dystrophy [BMD]) registered in the Chinese Genetic Disease Registry from March 2012 to August 2017 and analyzed the genetic mutational characteristics of these patients.

**Results:**

Large deletions were the most frequent type of mutation (72.2%), followed by nonsense mutations (11.9%), exon duplications (8.8%), small deletions (3.0%), splice-site mutations (2.1%), small insertions (1.3%), missense mutations (0.6%), and a combination mutation of a deletion and a duplication (0.1%). Exon 45–50 deletion was the most frequent deletion type, while exon 2 duplication was the most common duplication type. Two deletion hotspots were calculated—one located toward the central part (exon 45–52) of the gene and the other toward the 5’end (exon 8–26). We found no significant difference between hereditary and de novo mutations on deletion hotspots. Nonsense mutations accounted for 62.9% of all small mutations (197 patients).

**Conclusion:**

We built a comprehensive national dystrophinopathy mutation database in China, which is essential for basic and clinical research in this field. The mutational spectrum and characteristics of this DMD/BMD group were largely consistent with those in previous international DMD/BMD studies, with some differences. Based on our results, about 12% of DMD/BMD patients with nonsense mutations may benefit from stop codon read-through therapy. Additionally, the top three targets for exon-skipping therapy are exon 51 (141, 13.5%), exon 53 (115, 11.0%), and exon 45 (84, 8.0%).

**Electronic supplementary material:**

The online version of this article (10.1186/s13023-018-0853-z) contains supplementary material, which is available to authorized users.

## Introduction

*DMD* is the largest gene described in human beings, spanning more than 2.5 Mb of genomic sequence, and consisting of 79 exons. Mutations in *DMD* result in Duchenne muscular dystrophy (DMD) or Becker muscular dystrophy (BMD), collectively called dystrophinopathies. Mutations that disrupt the reading frame generally generate unstable RNA and lead to the production of nearly undetectable concentrations of truncated proteins, resulting in DMD. However, mutations always maintain the reading frame in BMD patients, resulting in truncated, but partly functional, dystrophin [1]. The reading frame rule applies to 90% of cases and is usually used both to confirm diagnosis of dystrophinopathies and distinguish DMD from BMD [2, 3].

The prevalence of dystrophinopathies is about one in 3600 to 6000 live male births [4]. DMD patients present with rapid deterioration of ambulation in early childhood, with boys usually losing the ability to walk before 12 years old. BMD patients show a milder course with patients preserving ambulation ability through 16 years of age. Clinicians make a diagnosis of Intermediate Muscular Dystrophy (IMD) for the intermediate phenotype.

Multiplex ligation-dependent probe amplification (MLPA) is performed in patients with related syndromes first since deletions and duplications are identified in the majority of patients through this method and the method is the most cost-effective approach to screen for these mutations. Patients who are MLPA-negative need further sequencing to detect small mutations [5].

Current care recommendations, such as glucocorticoids, cardiac protection, respiratory support, and rehabilitative functional training, can improve quality of life but cannot reverse the clinical course or prevent the inevitable outcome. Potential therapies focus on DNA/RNA-based approaches, such as viral vector-based gene therapy (DNA-based), gene-editing technology based on Clustered Regularly Interspaced Palindromic Repeats (DNA-based), stop codon read-through approach (RNA-based) and the exon-skipping approach (RNA-based). Ataluren (PTC-124) makes it possible to read through the premature stop codon and restore protein translation. Almost 83% of all DMD mutations may benefit from exon-skipping therapy [6].

The development of clinical trials in China for dystrophinopathies require more detailed information about mutation characteristics, natural history, and standards of clinical care, even though some hospital-based datasets, such as the Children’s Hospital of Fudan University database for dystrophinopathy in east China [7] and a comprehensive database in south China [8], already exist. Here, we analyzed genetic data of 1042 DMD/BMD patients based on a national registry database called “Chinese Genetic Diseases Registry” [9].

## Methods

### Patients and data collection

**Fig. 1:**
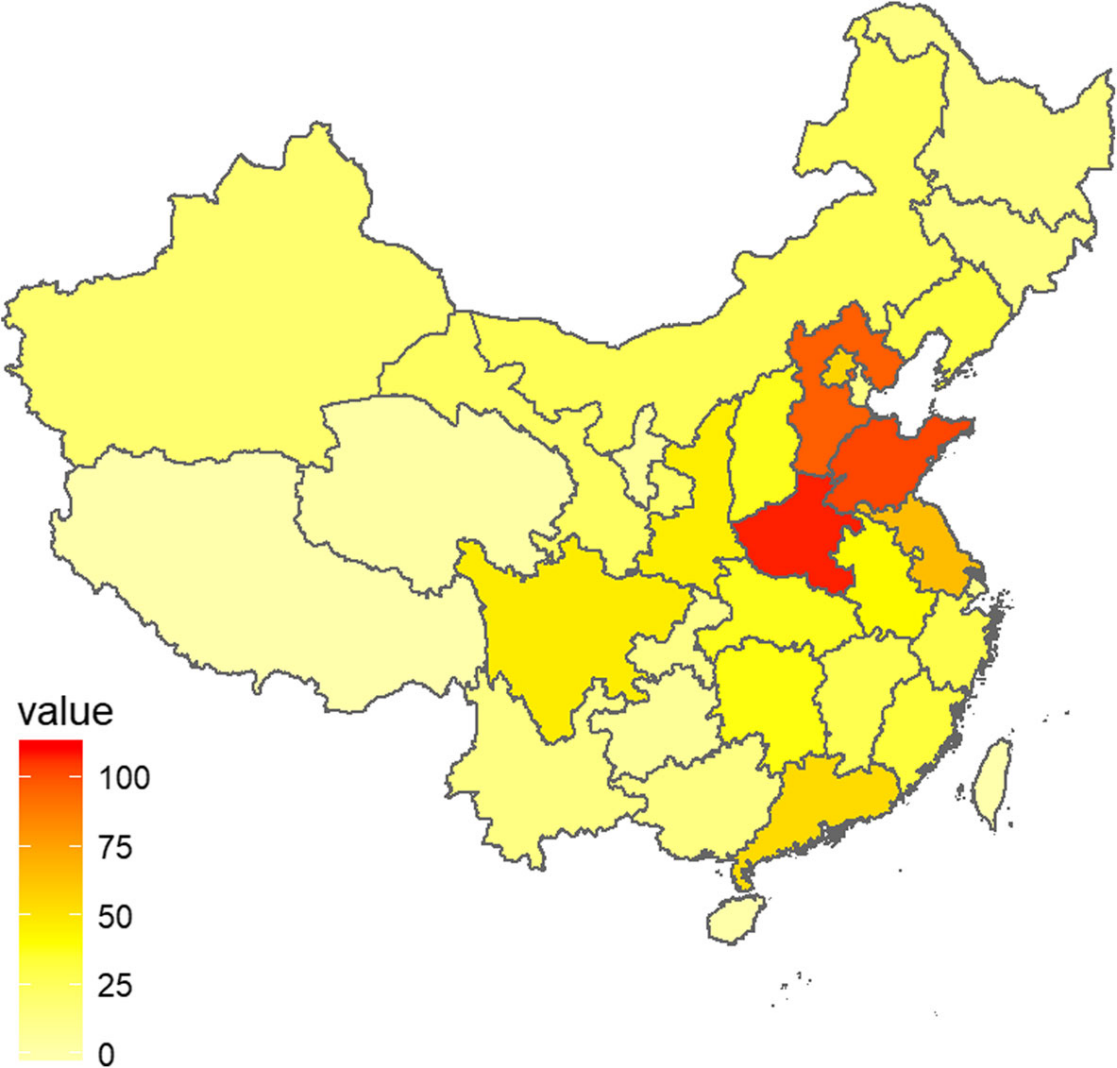
The geographical distribution of patients in China

We started the Chinese Genetic Disease Registry (www.dmd-registry.com) in 2012 and registered muscular diseases, including DMD, BMD, spinal muscular atrophy, and other neuromuscular disorders. More than 1400 DMD/BMD patients from all over China registered from inception through August 2017. Patients predominantly came from the eastern and central parts of China, probably due to the influence of geographic location, economic levels, and medical conditions (Fig. 1). Data collectors and analysts were hired to collect, collate, and upload data, follow-up by telephone, and perform other data collection and analytical tasks. Of all the DMD/BMD patients registered in the database, more than 500 patients took part in our multidisciplinary clinic. All data in the database will continue to be updated regularly at the patients’ outpatient visits or via telephone follow-up every 6 months.

### Diagnosis of dystrophinopathies

The diagnosis of DMD/BMD is confirmed by at least one of the following methods: (1) dystrophin protein deficiency demonstrated by muscle biopsy; (2) large deletion or duplication (≥1 exon) detected by MLPA; (3) small mutations, including nonsense mutations, missense mutation, splice-site mutations, small insertions, or deletions demonstrated by complete dystrophin gene sequencing. Computer software called “DMD toolkit” was developed to visualize the structure of *DMD* and to predict the functional changes of mutated dystrophin protein. In addition, the software helps improve the accuracy of clinical diagnosis [10].

## Results

More than 1400 DMD/BMD patients registered in our database. Using genetic testing, we confirmed 1042 cases of unrelated DMD/BMD. Patients who underwent only hotspot sequencing using multiple polymerase chain reactions were excluded from this study.

Among the mutations from these confirmed patients, 845 were large mutations (81.1%), of which 752 were large deletions (72.2% of all), 92 were large duplications (8.8% of all), and 1 was a combination mutation of a deletion and a duplication (0.1% of all). Of the 197 small mutations (18.9%), 124 were nonsense mutations (11.9% of all), 22 were splice-site mutations (2.1% of all), 31 were small deletions (3.0% of all), 14 were small insertions (1.3% of all), and six were missense mutations (0.6% of all). Of the confirmed cases, 863 (82.8% of all), 149 (14.3% of all), and 30 (2.9% of all) patients were diagnosed as DMD, BMD, and IMD, respectively.

### Frequency and hotspot distribution analysis of large mutations (deletion and duplication of ≥1 exon)

**Fig. 2:**
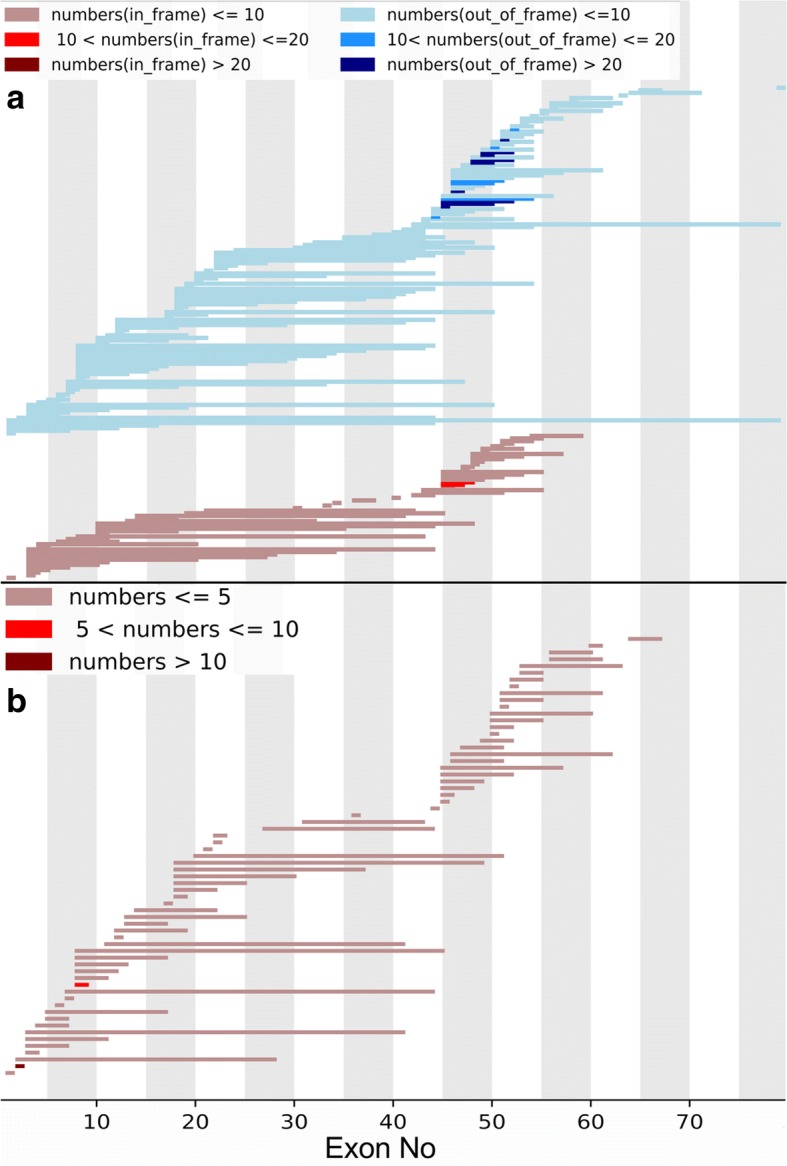
Patterns of exon deletion and duplication in *DMD* in DMD/BMD patients. **a** Exon deletion: each bar represents a type of exon deletion. **b** Exon duplication: each bar represents a type of exon duplication

**Fig. 3:**
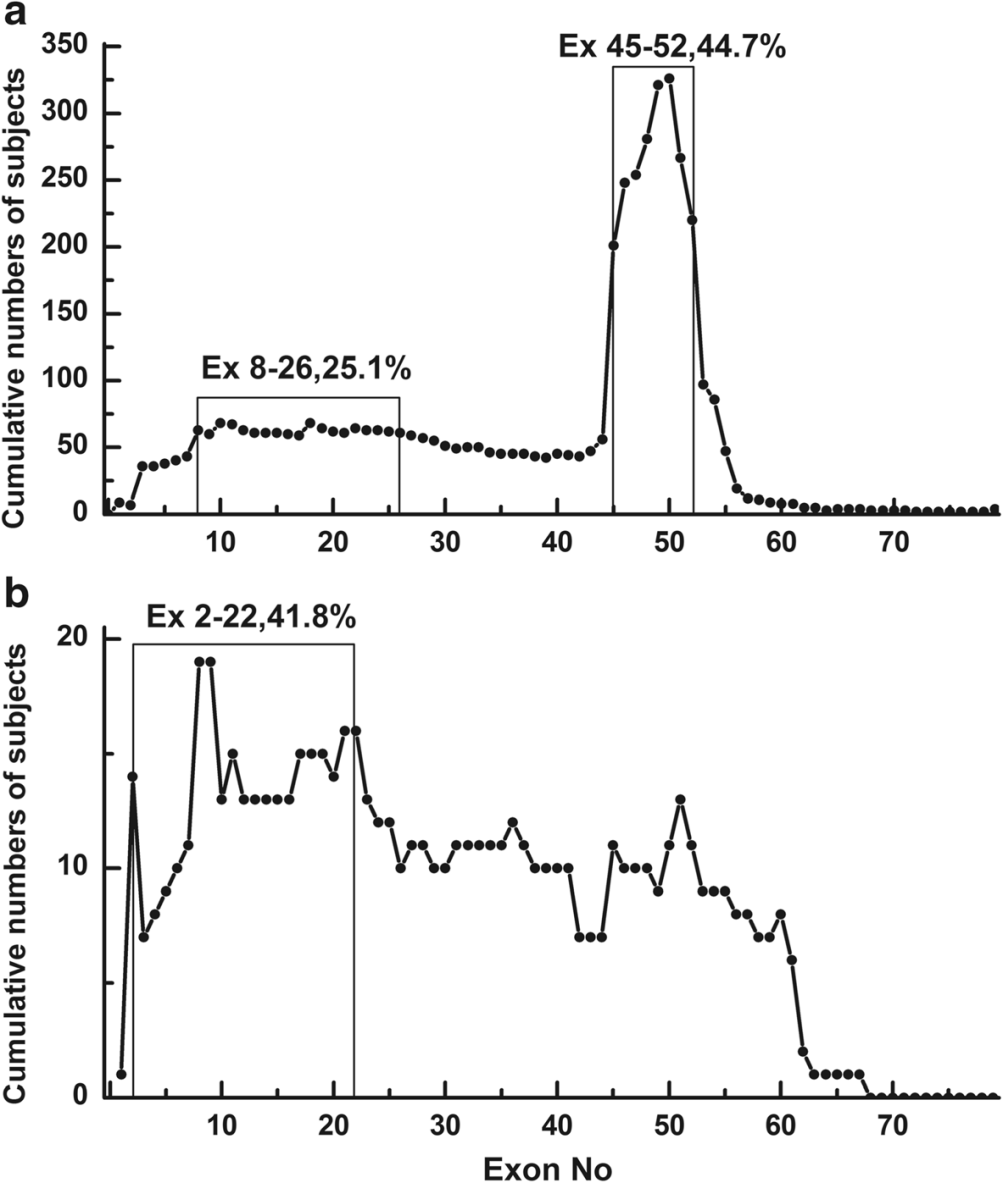
Cumulative numbers of subjects with deletion or duplication. **a** Deletions: a hotspot is visible between exon 45 and exon 52. **b** Duplications: a hotspot is visible between exon 2 and exon 22

The deletion and duplication frequency is depicted in Fig. 2a and b. The cumulative number of deletions and duplications is depicted in Fig. 3a and b. The exon 45–50 deletion (44/752, 5.9%) was the most frequent deletion type, and the exon 2 duplication (13/92, 14.1%) was the most common duplication type. Two deletion hotspots were observed: one located toward the central part of the gene and the other toward the 5′ end. The former was located in exons 45–52, which was the most common deletion region, containing up to 44.7% of all deletions. The latter hotspot included exons 8–26, taking up a smaller proportion (25.1%) of all deletions. A duplication hotspot was present between exon 2 and exon 22, making up 41.8% of all duplications.

The frequency of deletions starting in the central hotspot (exons 45–52) comprised 69.4% (522/ 752) of all deletions, while deletions starting in the proximal hotspot (exons 8–26) accounted for 12.5% (94/752) of all deletions. Large deletions affecting both hotspots were detected in seven patients (0.9%). Whole-gene deletion (exon 1–79) occurred in one patient. We found 188 different deletion types in the database. Of those, 54 deletion types were detected starting in the central hotspot region, while 59 deletion types started in the proximal hotspot, indicating that the proximal hotspot had greater diversity.

The frequency of duplications starting in the hotspot (exon 2–22) was as high as 64.1% (59/92). Duplications were more heterogeneous than deletions, with 66 types of duplication among 92 patients, 55 of which were reported only once in our database.

Two complex rearrangements were reported in our database: one patient held duplications in two different regions (exons 45–48 and exons 56–61), and the other patient harbored both a deletion and a duplication (exon 1 deletion and exon 2 duplication).

### Small mutations

**Fig. 4:**
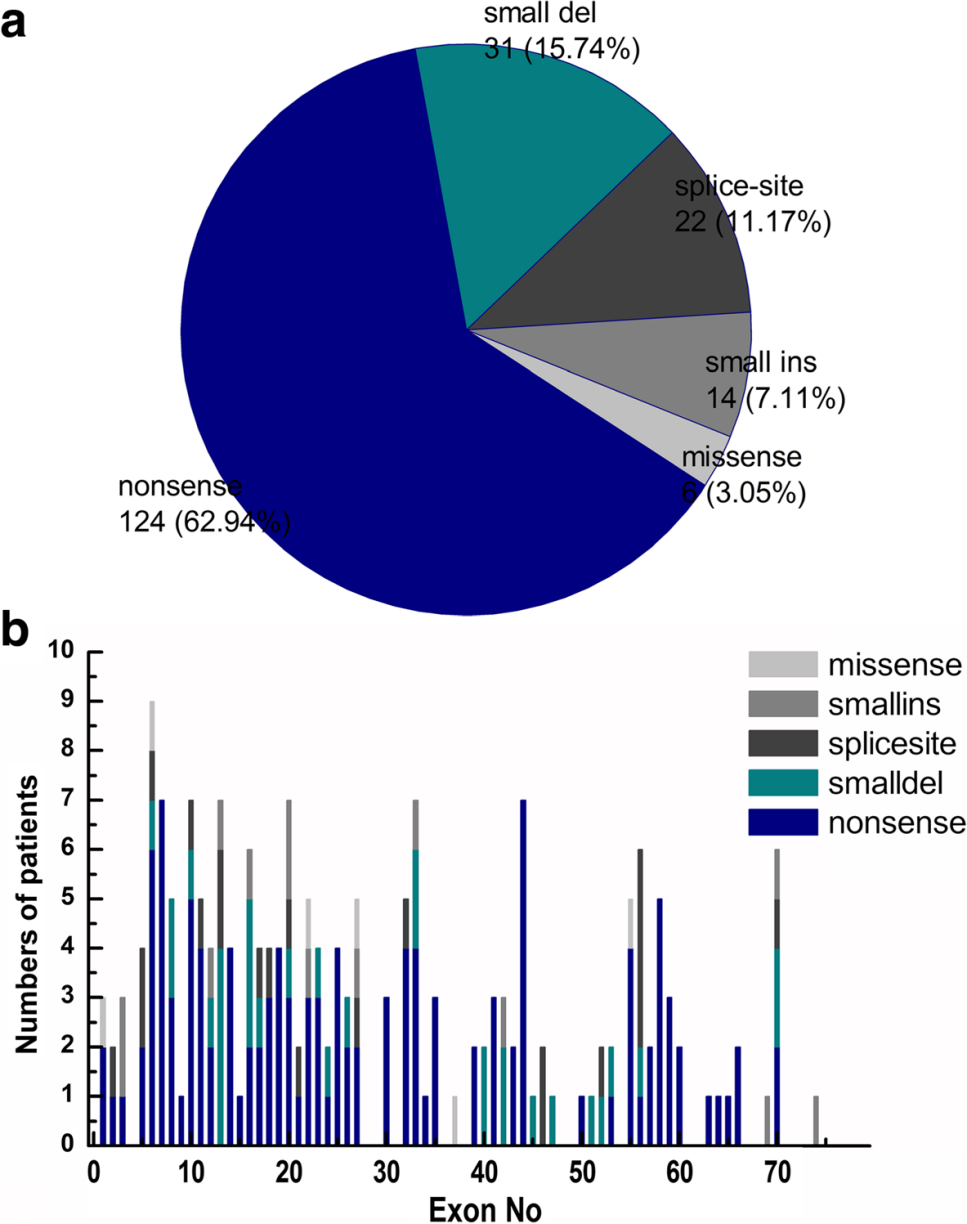
**a** Small mutation spectrum in dystrophinopathy patients. **b** Distribution of small mutations in *DMD*

The 197 small mutations represented 18.9% of all mutations in our database and consisted of 124 nonsense mutations (62.9%), 22 splice-site mutations (11.2%), 31 small deletions (15.7%), 14 small insertions (7.1%), and six missense mutations (3.0%) (Fig. 4a). Small mutations were varied and almost uniformly distributed throughout *DMD* (Fig. 4b). Only 109 of the 197 small mutations were reported according to the Leiden Open Variation Database [11]. Details of the small mutations is contained in Additional file 1. Of the 124 patients with nonsense mutations, two were clinically diagnosed with BMD, 13 patients with IMD, and 109 patients with DMD. Although nonsense mutations were almost evenly distributed throughout *DMD*, some types of nonsense mutation appeared more frequently. c.433c > T, c.583C > T, c.8608C > T and c.2302C > T were detected in 5, 5, 4, and 3 patients, respectively.

### Carrier state analysis

**Fig. 5:**
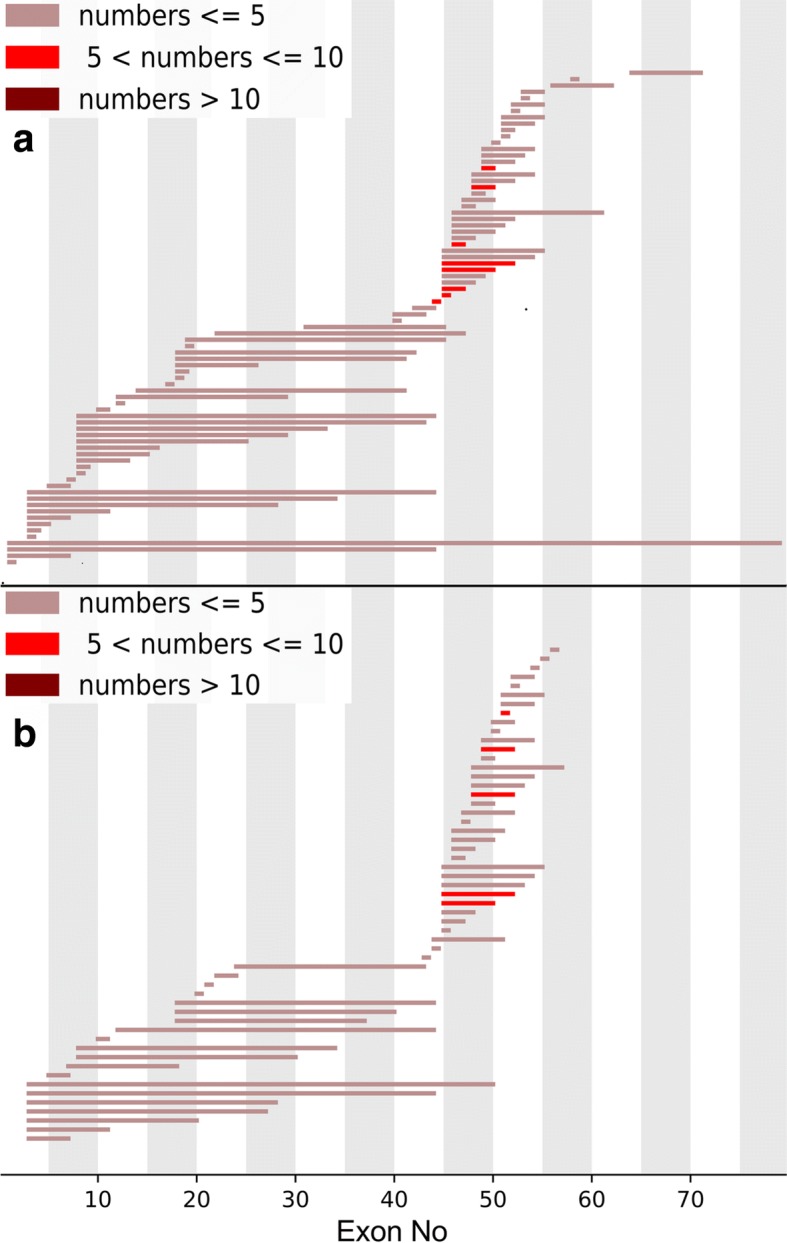
Patterns of exon deletion in *DMD* in the hereditary group and de novo group. **a** Exon deletion in the hereditary group: each bar represents a type of exon deletion. **b** Exon deletion in the de novo group: each bar represents a type of exon deletion

**Fig. 6:**
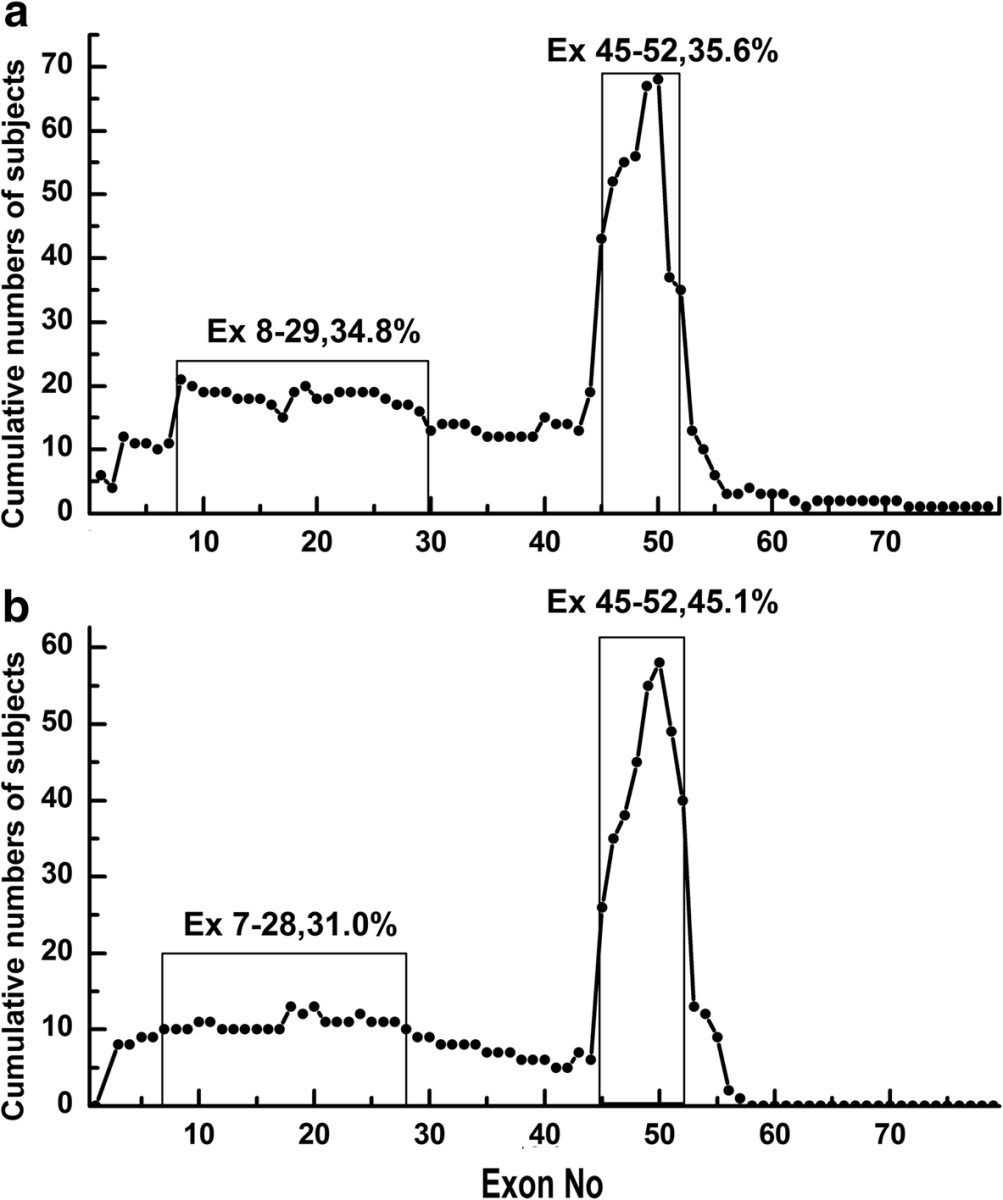
Cumulative numbers of subjects with deletions in the hereditary group and the de novo group. **a** Deletions in the hereditary group. A hotspot is visible between exon 45 and exon 52. **b** Deletions in the de novo group. A hotspot is visible between exon 45 and exon 52

Genetic analysis was performed on mothers of 442 probands. Of those, 297 (67.2%) possessed the same mutations as their children, while 145(32.8%)did not (Table 1). We analyzed deletion types and hotspot regions in the hereditary group and the de novo group, and the results are depicted in Figs. 5a, b, 6a, and b. We found no significant difference on deletion types and hotspot regions between the two groups, and the results were consistent with the deletion mutation distribution described above.

**Table 1:** Carrier state analysis of mothers of 442 probands

Mutation type	De novo	Hereditary	Total	Carrier rate
deletion	115	171	286	0.60
duplication	6	36	42	0.86
nonsense	16	57	73	0.78
small del	5	15	20	0.75
small ins	0	3	3	1.00
splice site	2	13	15	0.87
missense	1	2	3	0.67
Total	145	297	442	0.67

## Discussion

Analysis of dystrophin mutations and their distribution could reveal potential targets for gene therapies. In this study, we analyzed the mutational characteristics of a group of Chinese DMD/BMD patients based on a large comprehensive database. The results of our analysis of the mutation spectrum or carrier state were consistent with the findings of other studies with slight differences [7, 8, 12–17].

### Mutation distribution

Large mutations were the most prevalent mutation in many databases reported. Large and small mutations in this database were 81 and 19%, respectively, which was similar to those in the Leiden database (79%/21% [12]), the TREAT-NMD DMD database (80%/20% [13]), and the French UMD database (77.7%/22.3% [14]). However, compared with our results, some of the previous studies from China demonstrated lower large mutation rates and higher small mutation rates [8, 15, 18]. For example, Dan-Ni et al. reported the rates of large and small mutations in 132 patients were 68.7%/31.3% [8], respectively, which may be due to a smaller number of patients and the geographic concentration of registrants.

The most common mutations in *DMD* were intragenic deletions, which accounted for 65% of the dystrophin mutations [19]. In our database, large deletions (72.2%) and small deletions (3.0%) accounted for 75.2% of all mutations. The most frequent deletion hotspot region in our database was between exon 45 and exon 52 (44.7%), followed by the region between exon 8 and exon 26 (25.1%). The results are consistent with those in other studies [7, 12, 15–17, 19]. Two hotspots were located in the central part of the gene and in the 5′ end, though deletions can happen almost anywhere in *DMD* [19]. Some deletion types in hotspots were more frequently detected compared with others. For example, exon 45–50 (4.2% of all mutations) and exon 45 (3.5% of all mutations) deletions were the most common deletion types in this study, while exon 45 constituted 4% of all mutations in the TREAT-NMD DMD database and 2% in the Leiden database. As we expected, exon 2–22 and exon 2 were duplication hotspots and the most common exon duplication pattern in this study and previous studies, respectively [12–14, 16].

Hotspot regions and most common mutation types (duplication, deletion) were similar worldwide, which suggests that the proportion of exon deletions and duplications in DMD/BMD had minimal variance based on ethnicity [16, 20] and that some dystrophin gene regions are vulnerable to rearrangement [21]. However, this issue remains a matter of controversy [22–24].

### Carrier state analysis

Determination of carrier status is essential for both carrier screening and timely genetic counseling. Several DMD pedigrees contain more than one patient due to lack of knowledge about genetic counseling and prenatal diagnosis. Current genetic counseling practice is to attribute a carrier risk of two-thirds to the mother of a DMD patient [25]. In this study, we confirmed that the carrier rate for the mothers is 67%. The carrier rate of deletion mutations was 60%, while that of the other classes of mutations ranged from 75 to 86% (Table 1). Our data was also consistent with Haldane’s theoretical model of de novo mutations in X-linked diseases, as well as research in this field [26, 27], even though several smaller number studies showed that the carrier rate is much lower than the expected theoretical value [28–30].

Carrier state analysis revealed that exon 45–50 deletion was the most frequent deletions in both the carrier group (9/171, 5.3%) and the de novo group (8/115, 7.0%). Carrier state analysis also revealed a hotspot region located between exon 45 and exon 52, which was in approximately the same location as that of the whole study group. That suggests that no crucial difference exists between hereditary and de novo mutations.

### Potential therapies

RNA-based therapies, such as stop codon read-through therapy and exon-skipping therapy, give hope to patients with nonsense mutations and large deletion mutations. Ataluren (Translarna™) enables read-through of premature stop codons in mRNA to produce full-length and functional dystrophin protein and had been conditionally approved by the European Medicines Agency (EMA) for the treatment of DMD patients with nonsense mutation. Of the patients in this study, 11.9% may benefit from this therapy, 10% in the TREAT-NMD DMD Global database, 9.0% in the French UMD database, 10.5% in the Leiden database, and 13% in the Remudy database [12–14, 16]. All this data indicates that read-through therapy has significant potential in a wide range of clinical applications worldwide. Antisense oligonucleotide (AON)-mediated exon-skipping is another potential therapy for DMD patients that aims to produce partly functional proteins [31, 32]. Eteplirsen, used for exon 51 skipping, has been conditionally approved by the FDA and is now in clinical trials [33, 34]. Meanwhile, clinical trials targeting exon 53 and exon 45 skipping were recently initiated [35]. However, exon 51, 53, and 45 skipping would cumulatively account for 32.5% of all patients in our database (Table 2). AONs targeting additional exons are still in the developmental phase and face many challenges [32, 36]. Thus, international registries providing detailed data is crucial to address these challenges.

**Table 2:** The applicability of exon 51, exon 53, and exon 45 skipping for patients with deletion mutation

Skipped exon	Deleted exon	Number of patients
51	50	15
	52	20
	17–50	1
	3–50	1
	30–50	1
	45–50	44
	47–50	2
	48–50	26
	49–50	31
		total 141 (18.8% of all)
53	52	20
	43–52	1
	45–52	30
	47–52	3
	48–52	30
	49–52	26
	50–52	5
		total 115 (15.3% of all)
45	44	18
	46	1
	12–44	2
	18–44	2
	46–47	26
	46–48	8
	46–49	8
	46–51	15
	46–55	2
	46–57	2
		total 84 (11.2% of all)

## Conclusion

The database for dystrophinopathies we created is a registry containing a wealth of information about patients with DMD/BMD, including mutation characteristics, family history, epidemiological data, natural history, motor function, cardiac function, respiratory function, management status, and survival time. Our analysis of the data collected thus far revealed a mutational distribution in this Chinese group largely consistent with that found in previous reports. This database provides a reference for basic research, facilitates clinical trials, and promotes the development of future gene therapy.

## Additional file

Additional file 1: Clinical and genetic information of patients with small mutations. (PDF 93 kb)

## Abbreviations

BMD: Becker muscular dystrophy
CGDR: Chinese Genetic Disease Registry
CHFU: Children’s Hospital of Fudan University
DMD: Duchenne muscular dystrophy
IMD: Intermediate muscular dystrophy
LOVD: Leiden Open Variation Database
LSDBs: Locus specific databases
MLPA: Multiplex Ligation-dependent Probe Amplification
Remudy: Registry of Muscular Dystrophy
TREAT-NMD: Translational Research in Europe-Assessment and Treatment of Neuromuscular Diseases
