# Correction to: Comprehensive genetic characteristics of dystrophinopathies in China

**DOI:** 10.1186/s13023-021-01853-x

**Published:** 2021-06-02

**Authors:** Peipei Ma, Shu Zhang, Hao Zhang, Siying Fang, Yuru Dong, Yan Zhang, Weiwei Hao, Shiwen Wu, Yuying Zhao

**Affiliations:** 1 Department of Neurology, the General Hospital of Chinese People’s Armed Police Force, Beijing, China; 2 Department of Magnetic Resonance, the General Hospital of Chinese People’s Armed Police Force, Beijing, China; 3 Department of Precision Medicine Laboratory, the General Hospital of Chinese People’s Armed Police Force, Beijing, China; 4 Research Institute of Neuromuscular and Neurodegenerative Diseases and Department of Neurology, Qilu Hospital, Shandong University, Jinan, Shandong China

## Correction to: Ma et al. Orphanet Journal of Rare Diseases https://doi.org/10.1186/s13023-018-0853-z

The authors are grateful to the Editor of *Orphanet Journal of Rare Diseases* for allowing them the opportunity to publish this Correction, and apologize to the readership for any inconvenience caused.

A number of the variant descriptions in Additional file 1 of the original article [1] were not correct because of using different Refseq. The authors have now revised all the variant descriptions by Reference Sequence Database (NM_004006.2) in “Additional file 1” accompanying this Correction, as well as the original article.

All the authors agree to this Correction.

## Supplementary Information

**Additional file 1**. Clinical and genetic information of patients with small mutation.

## References

[1] Ma (2018). Comprehensive genetic characteristics of dystrophinopathies in China. Orphanet J Rare Dis.

