# Supplementary material for: Comprehensive genetic characteristics of dystrophinopathies in China
Source: Orphanet J Rare Dis. 2018 Jul 4;13:109. doi: 10.1186/s13023-018-0853-z (PMC6032532; doi:10.1186/s13023-018-0853-z)
Supplement: Supplementary file 1 — Clinical and genetic information of patients with small mutations. (PDF 93 kb) [file 13023_2018_853_MOESM1_ESM.pdf]

Additional file 1：Clinical and genetic information of patients with small mutation.

Patient	Age	Diagnosis	Ambulatory	Age at loss of ambulation	Mutation type	Exon	DNA change	Protein(predicted)	Report	Carrier status of mother
1	11.8	DMD	Yes		Nonsense	44	c.6370G>T	p.Glu2124*	unreported	Unknown
2	22.6	DMD	No	11.0	Nonsense	41	c.5889C>T	p.Arg1967*	reported	Yes
3	8.4	DMD	Yes		Nonsense	8	c.829C>T	p.Gln277*	reported	Yes
4	11.6	DMD	Yes		Small del	40	c.5602_5605delAGAA	p.Arg1868Glufs*5	reported	Unknown
5	12.9	DMD	No	9.0	Nonsense	55	c.8038C>T	p.2680Arg*	reported	Yes
6	8.7	DMD	Yes		Splicing	int32	c.4518+2T>C		reported	Yes
7	13.0	DMD	No	11.0	Small del	10	c.1076delA	p.Glu359Glyfs*17	unreported	Unknown
8	14.2	DMD	No	12.0	Nonsense	65	c.9445C>T	p.Gln3149*	reported	Unknown
9	10.4	DMD	Yes		Nonsense	59	c.8713C>T	p.Arg2905*	reported	Unknown
10	9.1	DMD	Yes		Nonsense	10	c.998C>A	p.Ser333*	reported	Yes
11	9.1	DMD	Yes		Nonsense	12	c.1465C>T	p.Gln489*	reported	Yes
12	7.6	DMD	Yes		Small del	56	c.8227delG	p.Gly2743Valfs*21	unreported	Yes
13	9.9	DMD	Yes		Nonsense	8	c.826C>T	p.Gln276*	reported	Unknown
14	13.3	DMD	No	9.0	Small del	6	c.522_523delTA	p.His174Glnfs*3	unreported	No
15	12.7	DMD	No	9.0	Nonsense	22	c.2832T>G	p.Tyr944*	unreported	Unknown
16	11.3	DMD	Yes		Nonsense	23	c.3087G>A	p.Trp1029*	reported	Yes
17	8.7	DMD	Yes		Nonsense	6	c.433c>T	p.Arg145*	reported	Unknown
18	8.1	DMD	Yes		Splicing	56	c.8390G>A		unreported	Unknown
19	6.0	DMD	Yes		Nonsense	58	c.8608C>T	p.Arg2870*	reported	Yes
20	9.5	DMD	Yes		Splicing	int17	c.2169-2A>G		reported	Unknown
21	9.0	DMD	Yes		Small del	12	c.1412delC	p.Thr471Lysfs*16	unreported	Yes
22	8.2	DMD	Yes		Nonsense	27	c.3622C>T	p.Gln1208*	reported	Unknown
23	8.9	DMD	Yes		Small del	70	c.10101_10103delAG	p.Glu3367del	reported	No
24	10.5	DMD	Yes		Nonsense	30	c.4117C>T	p.Gln1373*	reported	Unknown
25	12.5	IMD	Yes		Nonsense	5	c.336G>A	p.Trp112*	reported	Yes
26	14.0	DMD	No	7.0	Nonsense	59	c.8812G>T	p.Glu2938*	unreported	Unknown
27	10.6	DMD	Yes		Nonsense	56	c.8475G>A	p.Trp2915*	unreported	Unknown
28	12.0	DMD	No	9.0	Small del	45	c.6439-104253_6615-13966del	p.Glu2147Alafs*17	reported	Unknown
29	9.0	DMD	Yes		Nonsense	70	c.10108C>T	p.Arg3370*	reported	Unknown
30	10.1	DMD	Yes		Nonsense	14	c.1684C>T	p.Gln562*	reported	Yes
31	9.2	DMD	Yes		Nonsense	8	c.748G>T	p.Glu250*	reported	No
32	14.4	DMD	No	11.0	Small del	13	c.1593delA	p.Glu531Aspfs*52	unreported	No
33	11.6	DMD	No	7.0	Nonsense	64	c.9337C>T	p.Arg3113*	reported	Unknown
34	9.0	DMD	Yes		Small del	53	c.7755delG	p.Trp2585Cysfs*12	unreported	Yes
35	10.1	DMD	Yes		Nonsense	6	c.433c>T	p.Arg145*	reported	Unknown
36	5.6	DMD	Yes		Nonsense	44	c.6423C>A	p.Tyr2141*	reported	No
37	10.1	DMD	Yes		Splicing	int2	c.44-1G>T		unreported	Yes
38	14.3	IMD	Yes		Small ins	20	c.2462-2463insA	p.Glu821Glufs*	unreported	Unknown
39	12.9	IMD	Yes		Nonsense	7	c.583C>T	p.Arg195*	reported	Unknown
40	13.0	IMD	Yes		Nonsense	70	c.10141C>T	p.Arg3381*	reported	Unknown
41	11.9	DMD	No	9.0	Nonsense	58	c.8608C>T	p.Arg2870*	reported	No
42	7.2	DMD	Yes		Small del	51	c.7341delA	p.Gln2447Hisfs*13	unreported	Yes
43	12.2	DMD	No	9.0	Nonsense	55	c.8038C>T	p.Arg2680*	reported	Unknown
44	7.2	DMD	Yes		Splicing	int70	c.10228+1G＞A		reported	Unknown
45	14.9	DMD	No	12.0	Nonsense	57	c.8420G>A	p.Trp2807*	reported	Unknown
46	7.7	DMD	Yes		Nonsense	25	c.3328G>T	p.Glu1110*	reported	Yes
47	6.7	DMD	Yes		Small del	23	c.2962delT	p.Ser988Leufs*16	unreported	Yes
48	9.7	DMD	Yes		Nonsense	66	c.9568C>T	p.Arg3190*	reported	Yes
49	9.5	DMD	Yes		Small del	16	c.1872delA	p.Lys624Asnfs*8	unreported	Unknown
50	9.4	DMD	Yes		Small del	13	c.1533-1536del	p.His512Trpfs*4	reported	Unknown
51	8.6	DMD	No	8.0	Nonsense	35	c.4870C>T	p.Gln1624*	reported	No
52	12.6	IMD	Yes		Nonsense	30	c.4142T>G	p.Leu1381*	unreported	No
53	9.8	DMD	Yes		Splicing	int10	c.1150-2A>G		unreported	Yes
54	10.5	DMD	Yes		Small ins	42	c.6077-6078insA	p.Asp2026Glufs*4	unreported	Unknown
55	11.1	DMD	Yes		Small ins	27	c.3772dupT	p.Cys1258Leufs*1	unreported	Unknown
56	6.6	DMD	Yes		Splicing	int11	c.1114-2A>T		unreported	Unknown
57	4.8	DMD	Yes		Nonsense	23	c.3151C>T	p.Arg1051*	reported	Unknown
58	7.1	DMD	Yes		Small del	33	c.4583delA	p.Gln1528Argfs*18	unreported	Unknown
59	4.3	DMD	Yes		Small del	26	c.3466-3467del	p.Val1156Lysfs*7	unreported	Yes
60	11.7	DMD	Yes		Small ins	33	c.4561-4562insA	p.Met1521Asnfs*2	unreported	Unknown
61	6.4	DMD	Yes		Nonsense	33	c.4567G>T	p.Glu1523*	unreported	Unknown
62	15.9	DMD	No	12.0	Nonsense	33	c.4660G>T	p.Glu1554*	unreported	Unknown
63	20.8	IMD	No	14.0	Nonsense	16	c.1843C>T	p.Gln615*	reported	Unknown
64	8.2	DMD	Yes		Small ins	3	c.130dupC	p.Leu44Profs*45	unreported	Unknown
65	7.3	DMD	Yes		Nonsense	32	c.4483C>T	p.Gln1495*	reported	Unknown
66	11.8	DMD	No	8.0	Splicing	int56	c.8390+1G>A		reported	Unknown
67	6.2	DMD	Yes		Nonsense	63	c.9249G>A	p.Trp3083*	unreported	No

68	10.4	DMD	Yes		Nonsense	32	c.4414C>T	p.Gln1472*	reported	Yes
69	8.9	DMD	Yes		Nonsense	26	c.3580C>T	p.Gln1194*	reported	Unknown
70	6.8	DMD	Yes		Nonsense	20	c.2527G>T	p.Glu843*	reported	Unknown
71	15.0	IMD	Yes		Nonsense	17	c.2006T>C	p.Val669*	unreported	Yes
72	10.8	DMD	No	7.0	Nonsense	41	c.5899C>T	p.Arg1967*	reported	Yes
73	14.0	IMD	Yes		Nonsense	21	c.2758c>T	p.Gln920*	reported	Unknown
74	13.3	DMD	No	9.0	Nonsense	16	c.1938T>A	P.Cys646*	unreported	Yes
75	8.5	DMD	Yes		Nonsense	12	c.1339C>T	p.Gly447*	unreported	Yes
76	9.6	DMD	Yes		Small del	17	c.2034delG	p.Thr679Glnfs*3	unreported	Yes
77	5.5	DMD	Yes		Nonsense	33	c.4618G>T	p.Glu1540*	reported	Unknown
78	9.9	DMD	Yes		Splicing	int46	c.6762+2T>A		unreported	Yes
79	9.1	DMD	Yes		Nonsense	7	c.583C>T	p.Arg195*	reported	No
80	8.5	DMD	Yes		Small del	24	c.3205delG	p.Val1069Phefs*3	unreported	Yes
81	9.9	DMD	Yes		Splicing	int27	c.3417+1G>T		unreported	Yes
82	4.3	DMD	Yes		Nonsense	7	c.583C>T	p.Arg195*	reported	Unknown
83	6.3	DMD	Yes		Nonsense	19	c.2302C>T	p.Arg768*	reported	Unknown
84	5.2	DMD	Yes		Nonsense	22	c.2869C>T	p.Gln957*	reported	No
85	3.0	DMD	Yes		Small ins	12	c1427-1428insGA	p.Glu476Lysfs*11	unreported	Unknown
86	5.7	DMD	Yes		Nonsense	11	c.1255C>T	p.Gln419*	reported	Yes
87	4.9	DMD	Yes		Nonsense	6	c.433C>T	p.Arg145*	reported	Unknown
88	4.3	DMD	Yes		Nonsense	50	c.7288G>T	p.Gly2430*	unreported	Yes
89	3.3	DMD	Yes		Nonsense	3	c.119A>T	p.Lys1707*	unreported	Yes
90	4.1	DMD	Yes		Missense	55	c.7828C>A	p.Pro2610Thr	unreported	Yes
91	12.1	DMD	No	10.0	Small del	16	c.1979delA	p.Lys660Argfs*17	unreported	No
92	2.3	DMD	Yes		Nonsense	60	c.9029C>G	p.Ser3010*	reported	Yes
93	5.8	DMD	Yes		Nonsense	19	c.2368C>T	p.Gln790*	reported	Unknown
94	7.0	DMD	Yes		Nonsense	58	c.8608c>T	p.Arg2870*	reported	Yes
95	2.7	DMD	Yes		Splicing	int5	c.334-1G>A		unreported	Yes
96	3.2	DMD	Yes		Small del	42	c.6006_6007del	p.Glu2003Asnfs*1	unreported	Unknown
97	1.8	DMD	Yes		Nonsense	19	c.2302C>T	p.Arg768*	reported	Yes
98	9.9	DMD	Yes		Splicing	int46	c.6742+3T>A		unreported	Yes
99	2.3	DMD	Yes		Nonsense	24	c.3259C>T	p.Gln1087*	reported	Yes
100	11.0	DMD	Yes		Nonsense	25	c.3426C>A	p.Cys1142*	unreported	Unknown
101	5.3	DMD	Yes		Missense	6	c.496G>T	p.Gly166cys	unreported	No
102	2.9	DMD	Yes		Splicing	int52	c7661-1G>A		reported	Yes
103	6.7	DMD	Yes		Nonsense	20	c.2605C>T	p.Gln869*	reported	Yes
104	7.8	DMD	Yes		Nonsense	44	c.6292C>T	p.Arg2098*	reported	Yes
105	9.6	DMD	Yes		Nonsense	22	c.2832T>A	p.Tyr944*	unreported	No
106	12.9	IMD	Yes		Nonsense	27	c.3769A>T	p.Lys1257*	unreported	Unknown
107	8.0	DMD	Yes		Nonsense	10	c.984C>T	P.Arg1662*	unreported	Yes
108	3.2	DMD	Yes		Nonsense	26	c.3580C>T	p.Gln1194*	reported	Yes
109	10.1	DMD	Yes		Nonsense	18	c.2260C>T	p.Arg754*	unreported	Yes
110	11.7	DMD	Yes		Nonsense	10	c.1062G>A	p.Trp354*	reported	Unknown
111	6.9	DMD	Yes		Nonsense	30	c.4108C>T	p.Gln1370*	reported	Unknown
112	10.6	DMD	Yes		Nonsense	9	c.853G>T	p.Gly285*	reported	Unknown
113	10.0	DMD	No	9.0	Nonsense	43	c.6256G>T	p.Glu2086*	unreported	Yes
114	5.3	DMD	Yes		Nonsense	57	c.8443C>T	p.Gln2815*	reported	No
115	6.8	DMD	Yes		Nonsense	55	c.8194A>T	p.Lys2732*	reported	No
116	9.7	DMD	Yes		Splicing	int5	c.358-2A>G		reported	Yes
117	17.1	BMD	Yes		Nonsense	53	c.7672C>T	p.Gln2558*	reported	No
118	4.8	DMD	Yes		Nonsense	7	c.541C>T	p.Gln181*	unreported	No
119	6.9	DMD	Yes		Nonsense	17	c.1997C>A	p.ser666*	unreported	Unknown
120	3.2	DMD	Yes		Nonsense	25	c.3061C>T	p.Gln1021*	unreported	Yes
121	12.5	IMD	Yes		Nonsense	5	c.336G>A	p.Trp112*	reported	Yes
122	11.0	DMD	Yes		Nonsense	7	c.583C>T	p.Arg195*	reported	Unknown
123	13.9	DMD	No	12.0	Nonsense	2	c.77A>G	p.Asn26*	reported	Yes
124	13.5	IMD	Yes		Nonsense	25	c.3028G>T	p.Glu1010*	unreported	Yes
125	5.6	DMD	Yes		Nonsense	32	c.4405C>T	p.Gln1469*	reported	Yes
126	1.8	DMD	Yes		Small del	42	c.5996_5999del	p.Thr1999Ilefs*2	unreported	Yes
127	11.2	DMD	Yes		Nonsense	39	c.5530C>T	p.Arg1844*	reported	Yes
128	16.3	IMD	No	13.0	Splicing	int21	c.2804-1G>T		reported	No
129	7.4	DMD	Yes		Nonsense	6	c.433C>T	p.Arg145*	reported	Yes
130	7.0	DMD	Yes		Nonsense	11	c.1305G>A	p.Ser443*	unreported	Unknown
131	10.8	DMD	Yes		Small del	13	c.1593delA	p.Glu531Aspfs*52	unreported	Yes
132	7.7	DMD	Yes		Small ins	13	c.1540dupG	p.val514Glyfs*5	reported	Unknown
133	7.1	DMD	Yes		Nonsense	44	c.6310G>T	p.Glu2104*	reported	Unknown
134	5.5	DMD	Yes		Nonsense	59	c.8713C>T	p.Arg2905*	reported	Unknown
135	4.7	DMD	Yes		Small del	16	c.1978_1979del	p.Lys660Glufs*59	reported	Unknown
136	9.6	DMD	Yes		Nonsense	18	c.2213C>G	p.Ser738*	unreported	Unknown
137	7.4	DMD	Yes		Nonsense	44	c.6436A>T	p.Lys2146*	unreported	Yes
138	7.8	DMD	Yes		Small del	40	c.5697del	Lys1899Asnfs*2	reported	Yes

139	2.7	DMD	Yes		Missense	27	c.3774C>G	p.Cys1258Trp	unreported	Yes
140	5.2	DMD	Yes		Nonsense	14	c.1702C>T	p.Gln568*	reported	Yes
141	5.5	DMD	Yes		Nonsense	44	c.6292C>T	p.Arg2098*	unreported	Yes
142	2.6	DMD	Yes		Nonsense	66	c.9568C>T	p.Arg3190*	reported	Yes
143	2.8	DMD	Yes		Nonsense	7	c.559C>T	p.Gln187*	unreported	Yes
144	8.1	DMD	Yes		Splicing	int56	c.8300-IG>C		unreported	No
145	9.7	DMD	Yes		Nonsense	15	c.1777C>T	p.Gln593*	reported	No
146	9.1	DMD	Yes		Small del	8	c.676-678delAAG	p.lys226del	reported	No
147	12.2	IMD	Yes		Small ins	3	c115-117 insA	p.Asn39Lysfs *5	unreported	Unknown
148	9.3	DMD	Yes		Nonsense	44	c.6373c>T	p.Gln2125*	reported	Yes
149	14.8	DMD	No	8.0	Small del	47	c.6807-6810delACAA	p.Leu2270Metfs*9	unreported	Unknown
150	11.0	DMD	Yes		Nonsense	55	c.8215C>T	p.Gln2739*	unreported	Unknown
151	9.7	DMD	Yes		Nonsense	6	c.436C>T	p.Gln146*	reported	Yes
152	10.0	DMD	Yes		Small del	52	c.7559delT	p.Leu2520Trpfs*18	unreported	Yes
153	12.6	IMD	Yes		Small del	20	c.2471_2474del	p.Asn824Serfs*21	unreported	Yes
154	9.9	DMD	Yes		Nonsense	34	c.4732A>T	P.Lys1578*	unreported	Unknown
155	11.9	DMD	No	8.0	Nonsense	58	c.8608C>T	p.Arg2870*	reported	Unknown
156	8.9	DMD	Yes		Missense	37	c.5234G>A	p.Arg1745His	reported	Unknown
157	8.5	DMD	Yes		Small ins	16	c.1898dupA	Asn633Lysfs*2	unreported	Yes
158	11.5	DMD	Yes		Small del	70	c10101_10103del	p.Glu3367del	reported	Yes
159	7.3	DMD	Yes		Missense	1	c.1A>T	p.Met1Leu	unreported	Unknown
160	10.3	DMD	Yes		Small ins	70	c.1648dupC	p.Arg550Profs*17	unreported	Yes
161	9.4	DMD	Yes		Small del	13	c.1533_1536del	His512Trpfs*4	reported	Unknown
162	10.2	DMD	Yes		Small ins	69	c.10027_10028insT	p.Ser3343Phefs*9	unreported	Unknown
163	10.7	DMD	Yes		Splicing	int56	c.8391-1G>A		reported	Yes
164	12.8	IMD	Yes		Missense	22	c.2949G>T	p.Gln983His	reported	Unknown
165	6.2	DMD	Yes		Nonsense	41	c.5851C>T	p.Gln1951*	reported	Unknown
166	10.3	DMD	Yes		Small ins	74	c.10453-10454insC	p.Leu3485Profs*6	unreported	Unknown
167	13.1	IMD	Yes		Nonsense	14	c.1702C>T	p.Gln568*	reported	Yes
168	7.1	DMD	Yes		Nonsense	10	c.998C>A	p.Ser333*	reported	Yes
169	10.6	DMD	Yes		Nonsense	14	c.1638G>A	p.Trp546*	reported	Yes
170	24.7	BMD	Yes		Splicing	int18	c.2254-2A>T		unreported	Yes
171	6.0	DMD	Yes		Small del	8	c.676-678delAAG	p.Lys226del	reported	Unknown
172	12.9	IMD	Yes		Splicing	int20	c.2435-2A>T		unreported	Unknown
173	11.0	DMD	Yes		Small ins	20	c.2462-2463insA	p.Glu821Glufs*4	unreported	Unknown
174	10.9	DMD	Yes		Nonsense	23	c.3034delC	p.Leu1012*	unreported	Unknown
175	7	DMD	Yes		Nonsense	11	c.1305G>A	p.Ser443*	unreported	No
176	12.0	IMD	Yes		Nonsense	43	c.6223C>T	p.Gln2075*	reported	No
177#	6.6	DMD	Yes		Splicing	int13	c.1114-1 G>A*		unreported	Yes
178#	6.6	DMD	Yes		Splicing	int13	c.1114-1 G>A*		unreported	Yes
179	10.9	DMD	Yes		Small ins	22	c.2864dupT	Gln956Profs*13	unreported	Yes
180	13.9	IMD	Yes		Nonsense	20	c.2518G>T	p.Gln840*	reported	Unknown
181	8.3	DMD	Yes		Splicing	int6	c.531-2A>G		unreported	Unknown
182	37.5	BMD	Yes		Nonsense	1	c.11G>A	p.Trp4*	reported	Unknown
183	4.6	DMD	Yes		Nonsense	1	c.12G>A	p.Trp4*	unreported	Yes
184	6.3	DMD	Yes		Nonsense	19	c.2302C>T	p.Arg768*	reported	Yes
185	5.7	DMD	Yes		Nonsense	11	c.1255C>T	p.Gln419*	unreported	Yes
186	8.9	DMD	Yes		Nonsense	60	c.8944C>T	p.Arg2982*	reported	Yes
187	4.9	DMD	Yes		Nonsense	6	c.433C>T	p.Arg145*	reported	Yes
188	6.0	DMD	Yes		Nonsense	58	c.8608C>T	p.Arg2870*	reported	Yes
189	11.2	DMD	Yes		Nonsense	39	c.5530C>T	p.Arg1844*	reported	Unknown
190	7.3	DMD	Yes		Nonsense	35	c.4996C>T	p.Arg1666*	reported	Unknown
191	6.4	DMD	Yes		Nonsense	35	c.4561G>T	p.Glu1523*	unreported	Yes
192	5.5	DMD	Yes		Nonsense	33	c.4618G>T	p.Glu1540*	reported	Yes
193	10.1	DMD	Yes		Nonsense	18	c.2260C>T	p.Arg754*	unreported	Yes
194	11.0	DMD	Yes		Nonsense	32	c.4375C>T	p.Arg1459*	reported	Unknown
195	8.0	DMD	Yes		Nonsense	10	c.984C>T	p.Arg1662*	unreported	Yes
196	4.3	DMD	Yes		Nonsense	7	c.583C>T	p.Arg195*	reported	Yes
197	9.2	DMD	No	9.0	Small del	33	c.4587delG	p.Lys1529Asnfs*13	reported	Yes

The patients with # are brothers.
